# Treatment period and changes in bone markers according to the application of teriparatide in treating medication-related osteonecrosis of the jaw

**DOI:** 10.1186/s12903-025-05867-w

**Published:** 2025-04-11

**Authors:** Jin Hoo Park, Wonse Park, Loi Phuoc Nguyen, Jin-Woo Kim, Sanghuem Cho, Hyunmi Jo, Hyung Jun Kim, Young-Soo Jung, Jun-Young Kim

**Affiliations:** 1 Department of Oral & Maxillofacial Surgery, Yonsei University College of Dentistry, Seoul, South Korea; 2 Department of Advanced General Dentistry, Yonsei University College of Dentistry, Seoul, South Korea; 3 Institute for Innovation in Digital Healthcare, Yonsei University, Seoul, South Korea; 4 Department of Maxillofacial Surgery, Faculty of Odonto-Stomatology, University of Medicine and Pharmacy at Ho, Chi Minh City, Vietnam; 5 Department of Oral and Maxillofacial Surgery, College of Medicine, Ewha Womans University, Seoul, South Korea; 6 Department of Oral and Maxillofacial Surgery and Oral Science Research Center, Yonsei University College of Dentistry, 50-1 Yonsei-ro, Seodaemun-gu, Seoul, 03722 Korea

**Keywords:** Teriparatide, Medication-related osteonecrosis of the jaw, Treatment period, Changes in bone markers

## Abstract

**Background:**

This study aimed to explore the effects of teriparatide (TPTD) on treatment duration, surgical procedures, and bone turnover markers in medication-related osteonecrosis of the jaw (MRONJ).

**Methods:**

We analyzed 76 patients with MRONJ post-treatment and divided them into conservative/surgical and TPTD/non-TPTD groups. Key assessments included treatment duration, surgery count, and changes in bone markers (serum C-terminal telopeptide of type 1 collagen [CTX], osteocalcin [OC], procollagen type 1 N-terminal propeptide [P1NP], parathyroid hormone [PTH], 25-OH-vitamin D [25(OH)D], calcium, and inorganic phosphorus) measured at the initial and post-treatment stages.

**Results:**

TPTD-treated surgical patients experienced shorter treatment periods and underwent fewer surgeries than did non-TPTD counterparts. Post-treatment, both groups showed significant increases in CTX, OC, and 25(OH)D levels. P1NP elevation was significant only in the non-TPTD group. Although the PTH levels decreased in both groups, the difference was not statistically significant. Calcium and phosphorus levels increased in both groups, but only calcium levels increased significantly in the TPTD group. Additionally, TPTD-treated patients showed significant improvements in T-scores, particularly in the lumbar spine and femur neck, compared to the non-TPTD group.

**Conclusions:**

TPTD administration during MRONJ treatment potentially reduces the need for surgical intervention and accelerates recovery, significantly affecting bone metabolism. These findings highlight TPTD’s role in enhancing the efficacy of MRONJ treatment. TPTD could potentially offer the dual benefit of promoting bone healing and reducing the need for surgical intervention, thus improving overall outcomes for patients with MRONJ.

## Background

Bisphosphonates and denosumab are commonly used to prevent fractures in osteoporosis treatment [1]. Bisphosphonates act on the receptor activator of nuclear factor kB ligand, an osteoblast differentiation factor that inhibits the activity of both osteoblasts and osteoclasts in the bone. This suppression of bone resorption, limitation of skeletal remodeling, and increase in bone density have notable side effects [2]. An adverse side effect is “medication-related osteonecrosis of the jaws (MRONJ),” characterized by necrosis of the jawbone within the oral cavity.

MRONJ, first described by Marx in 2003 [3], is defined by the American association of oral and maxillofacial surgeons (AAOMS) as “a condition in which necrotic bone of the jaw persists for more than 8 weeks in patients who have not received prior radiation therapy to the head and neck area and who are currently receiving or have previously received anti-resorptive medication.” The pathogenesis of MRONJ has not yet been clearly elucidated. However, several hypotheses exist, the main ones being bone remodeling inhibition, inflammation or infection, angiogenesis inhibition, innate or acquired immune dysfunction, and genetic predisposition. With more studies being conducted, it is becoming increasingly evident that MRONJ is a multifactorial disease [4].

Studies on MRONJ treatment methods are crucial for oral and maxillofacial surgeons [5]. According to the AAOMS, MRONJ treatment promotes natural healing through continuous antibiotic therapy and oral disinfection, recommending the discontinuation or replacement of causative drugs [4]. Surgical intervention is advised when conservative treatment fails or in cases of extensive osteonecrosis.

Additionally, studies on adjunctive treatment methods, including hyperbaric oxygen therapy, ozone therapy, pentoxifylline, vitamin E, and teriparatide (TPTD) injections, are ongoing [6–8]. Particularly, TPTD, first attempted by Harper in 2007, is a recombinant form of parathyroid hormone (PTH) [9]. Unlike bisphosphonates, TPTD activates osteoblasts and enhances the metabolic function of osteoclasts to promote skeletal remodeling [2, 10].

Recent studies have also considered testing for serological bone turnover markers as an important factor in determining the onset and treatment prognosis of MRONJ [11, 12]. Controversially, several studies have focused on the serum C-terminal telopeptide cross-link between type 1 collagen (CTX) levels and MRONJ risk, suggesting that lower CTX levels increase the likelihood of developing MRONJ [12–14].

Given the interest in TPTD and bone marker tests in MRONJ onset, prediction, and treatment, most studies have reported positive outcomes with TPTD application in MRONJ treatment [8, 10, 15, 16]. However, there is a lack of comparative studies on MRONJ treatment outcomes with and without TPTD, and patient numbers in existing studies are insufficient [15]. Furthermore, evaluation of changes in bone marker values with and without TPTD application is crucial for assessing bone metabolism activity. Although several studies have examined the correlation between CTX and MRONJ, studies on the levels of other bone markers, their patterns of change before and after MRONJ treatment, and differences based on TPTD application are limited [14, 17, 18].

We aimed to determine whether preoperative TPTD application in the treatment of MRONJ could shorten the treatment period, improve treatment efficiency, and change bone activity. To this end, this study investigated the differences in the treatment period, bone marker values, ​​and T-scores before and after treatment between the groups administered TPTD and those not administered.

## Methods

### Study participants

From January 2010 to December 2021, 2,777 patients who visited the Department of Oral and Maxillofacial Surgery at Yonsei University Dental Hospital and underwent serum bone turnover marker testing were considered. This study included patients with the following characteristics:

- Patients with a history of osteoporosis (as indicated by a T-score of − 2.5 or lower or based on clinical assessment) and treated with bisphosphonate or denosumab.
- Were diagnosed with MRONJ at our hospital, treated, and completely cured.
- Underwent serum bone turnover marker tests, including CTX, osteocalcin (OC), procollagen type 1 N-terminal propeptide (P1NP), PTH, 25-OH-vitamin D, calcium, and inorganic P, during MRONJ treatment.

Patients with a history of head and neck radiation therapy and those taking antiresorptive agents for non-osteoporotic conditions were excluded. Seventy-six patients met the inclusion criteria: 16 in the conservative group and 60 in the surgical group.

This study was conducted in accordance with the Declaration of Helsinki and was approved by the Institutional Research Ethics Committee of Yonsei University College of Dentistry (IRB No. 2-2021-0017). The need for informed consent was waived as this study is a retrospective analysis of medical records and was deemed unnecessary according to national regulations, specifically Article 18 of the Bioethics and Safety Act of South Korea.

### Treatment process of medication-related osteonecrosis of the jaw (MRONJ)

When diagnosed with MRONJ, patients first received conservative treatment. Conservative treatment includes (1) broad-spectrum antibiotic therapy, (2) continuous oral dressing, (3) discontinuation and replacement of drugs causing MRONJ, and (4) treatment of TPTD. However, surgical treatment performed under local or general anesthesia was not included in conservative treatment. Drug replacement or discontinuation was decided upon through medical consultation after evaluating MRONJ-inducing drugs. According to our hospital protocol, patients with MRONJ were referred to the endocrinology department for drug holiday, TPTD treatment, and osteoporosis management. There they were prescribed 1,000 IU/day of Vitamin D orally for osteoporosis management. TPTD treatment (Forsteo^®^, 20 µg daily subcutaneous injection, up to 24 months) was performed by an endocrinologist after obtaining patient consent. TPTD treatment was terminated following resolution of MRONJ, and osteoporosis treatment was continued with other drugs when necessary. Patients were deemed fully cured when conservative treatment resulted in natural necrotic bone shedding and the disappearance of clinical and radiological symptoms with complete mucosal coverage and healing.

The decision to transition from conservative to surgical therapy was guided by various factors, including the duration of use and discontinuation period of antiresorptive drugs, increase in bone turnover marker levels, presence of sequestration, or lack of effective healing with conservative treatment. Surgical intervention included procedures such as sequestrectomy, curettage, tooth extraction, and implant removal. Additional surgeries were performed if recurrence signs such as inflammation, infection, or bone formation, necrosis or exposure persisted after the first surgery. Based on the condition of the lesion, simple excision including sequestrectomy and wide excision were performed. However, reconstruction surgery using flaps, etc. may be another factor of healing; thus, extensive surgery requiring reconstruction was excluded from this study.

In our study, end of treatment was defined by the complete resolution of clinical symptoms, such as absence of pain, infection, and swelling, as well as the complete coverage and healing of mucosal lesions with no bone exposure. Additionally, treatment was considered complete when there was no recurrence of the disease over a follow-up period. This was further confirmed by observing mucosal healing at least 2 months after surgery. In all the participants in this study, mucosal healing was achieved at 2 months after surgery without any additional complications.

### Grouping and data collection

The patients were categorized into four groups based on the treatment type and TPTD application. The TPTD group consisted of patients who received TPTD treatment for > 3 months.

- Conservative: Non-TPTD (*n* = 8).
- Conservative: TPTD (*n* = 8).
- Surgical: Non-TPTD (*n* = 33).
- Surgical: TPTD (*n* = 27).

The patients’ dental and medical charts were thoroughly reviewed to identify the characteristics and MRONJ-related risk factors. This review included the following:

1. Age, sex, time of first symptom onset, dental treatment before symptom onset, and location of onset.
2. Type, duration, and route of administration of MRONJ-related drugs (oral or intravenous).
3. Patient’s underlying diseases, such as hypertension, diabetes, rheumatic disease, and heart disease.
4. Oral clinical symptoms, including pain, pus discharge, gingival swelling, and numbness.
5. MRONJ stage, as per the 2022 AAOMS criteria [4].

The duration (in months) from the first visit to complete recovery was documented for each patient. In cases involving surgical procedures, the time (in months) from the start date of the surgery to complete recovery was also recorded. Additionally, the number of sequestrectomies or curettages performed under local or general anesthesia was noted. Levels of bone markers, including CTX, OC, P1NP, PTH, 25-OH-vitamin D, calcium, and inorganic P, were measured at the first visit and upon treatment completion.

4. Comparative analysis of T-score changes in TPTD and non-TPTD groups.

Data from patients who underwent dual-energy X-ray absorptiometry (DXA) to assess bone mineral density before and after MRONJ treatment were retrospectively analyzed. T-scores were obtained from the lumbar spine, femur neck, and hip using DXA scans (Hologic Discovery ATM, Hologic Inc., Bedford, MA, USA), which were centrally read and analyzed. Patients were divided into two groups based on whether they received TPTD treatment. The changes in T-scores before and after MRONJ treatment were compared to evaluate the effects of TPTD on bone density.

### Statistical methods

IBM SPSS statistics version 27 was used for statistical analyses. Recovery periods and bone marker levels (CTX, OC, P1NP, PTH, 25-OH-vitamin D, calcium, inorganic P) were evaluated at the first visit and treatment completion. Fisher’s exact test, one-way analysis of variance, Kruskal–Wallis analysis, Mann–Whitney analysis, and paired samples T-test were used for various comparisons.

## Results

### Comparison of MRONJ-related factors of patients in four groups

**Fig. 1:**
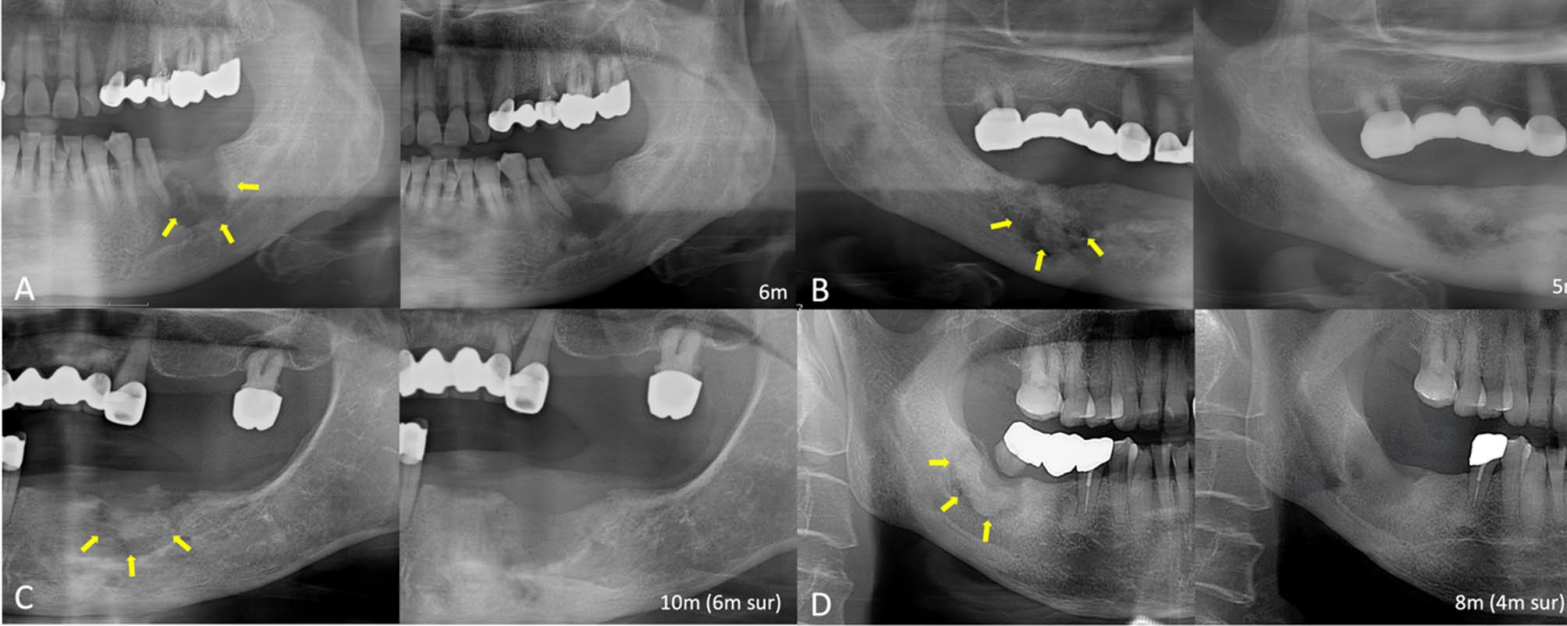
Panoramic view of medication-related osteonecrosis of the jaw (MRONJ) lesion before and after treatment. (**a**) non-TPTD conservative treatment (pre treatment(Tx.), after Tx. 6months), (**b**) TPTD conservative treatment (pre Tx., after Tx. 5months), (**c**) non-TPTD surgical treatment (pre Tx., after Tx. 10months, after surgery (Sx.) 6months), (**d**) TPTD surgical treatment (pre Tx., after Tx. 8months, after Sx. 4months). (Yellow arrows indicate margins of the necrotic bone in the MRONJ site) TPTD, teriparatide

Across the four groups, sex, medication use, location of occurrence, dental factors, clinical symptoms, underlying disease, and disease stage were analyzed (Table 1). In the conservative-only treatment group (*n* = 16), patients were divided into those who did and did not receive TPTD (*n* = 8 in each subgroup). The patients in the surgical group (*n* = 60) were similarly divided (TPTD, *n* = 27; non-TPTD, *n* = 33). Except for the location of occurrence, no significant differences in MRONJ-related characteristics were observed between the groups. The conservative group had a higher incidence of maxillary posterior involvement (50.0%), whereas the surgical group had a higher incidence of mandibular posterior involvement (66.7%), with a statistically significant difference (*p* = 0.006). Additionally, Fig. 1 includes representative examples of panoramic radiographs taken before and after treatment for each group, providing a visual representation of the treatment outcomes.

**Table 1:** Patient characteristics at baseline

		Conservative group (*n* = 16)		Surgical group (*n* = 60)			
Variables		**Non-TPTD (n = 8)**	**TPTD (n = 8)**		**Non-TPTD (n = 33)**	**TPTD (n = 27)**	**p-value**
Sex (female: male)		8:0	8:0		30:3	27:0	0.430
Age (mean ± SD) (years)		69.63 ± 8.83	74.75 ± 8.91		74.94 ± 7.26	77.11 ± 6.89	0.552
Medication (N, %)	Alendronate	5 (62.5%)	3 (37.5%)		6 (18.2%)	9 (33.3%)	0.588
	Ibandronate	2 (25.0%)	2 (25.0%)		13 (39.4%)	9 (33.3%)	
	Risedronate	0	1 (12.5%)		3 (9.1%)	2 (7.4%)	
	Zoledronate	0	0		1 (3.0%)	0	
	Pamidronate	0	0		0	1 (3.7%)	
	Denosumab	1 (12.5%)	0		2 (6.1%)	0	
	Unknown	0	2 (25.0%)		8 (24.2%)	6 (22.2%)	
Administered route	PO (N, %)	6 (75.0%)	8 (100%)		25 (75.8%)	24 (88.9%)	0.461
	IV (N, %)	1 (12.5%)	0		6 (18.2%)	3 (11.1%)	
	Subcutaneous (N, %)	1 (12.5%)	0		2 (6.1%)	0	
	PO_duration (Mean ± SD) (years)	4.67 ± 2.81	6 ± 2.83		4.92 ± 3.46	6.04 ± 1.31	0.731
	IV_duration (Mean ± SD) (years)	1 ± 0.00			2.7 ± 2.25	2 ± 1.00	0.669
	Subcutaneous_duration (Mean ± SD) (years)	1 ± 0.00			2 ± 0.00		0.083
Location (N, %)	Maxillary anterior	0	1 (12.5%)		0	0	0.03*
	Maxillary posterior	4 (50.0%)	4 (50.0%)		9 (27.3%)	6 (22.2%)	
	Mandibular anterior	1 (12.5%)	2 (25.0%)		2 (6.1%)	3 (11.1%)	
	Mandibular posterior	3 (37.5%)	1 (12.5%)		22 (66.7%)	18 (66.7%)	
Dental procedure (N, %)	Tooth related^**^	2 (25.0%)	3 (37.5%)		18 (54.5%)	16 (59.3%)	0.588
	Dental implant^***^	3 (37.5%)	3 (37.5%)		10 (30.3%)	7 (25.9%)	
	Edentulous ridge	3 (37.5%)	2 (25.0%)		5 (15.2%)	4 (14.8%)	
Symptom (N, %)	Pain	4 (50.0%)	4 (50.0%)		16 (48.5%)	19 (70.4%)	0.364
	Pus	4 (50.0%)	4 (50.0%)		16 (48.5%)	14 (51.9%)	1.000
	Gingival swelling	4 (50.0%)	3 (37.5%)		16 (48.5%)	14 (51.9%)	0.953
	Numbness	1 (12.5%)	0		0	2 (7.4%)	0.190
	Others	0	0		4 (12.1%)	4 (14.8%)	0.714
Comorbidity (N, %)	Hypertension	2 (25.0%)	6 (75.0%)		18 (54.5%)	15 (55.6%)	0.277
	Diabetes	3 (37.5%)	3 (37.5%)		7 (21.2%)	6 (22.2%)	0.567
	Cardiac disease	2 (25.0%)	2 (25.0%)		4 (12.1%)	2 (7.4%)	0.290
	Rheumatoid arthritis	0	1 (12.5%)		2 (6.1%)	1 (3.7%)	0.741
	Steroid	0	1 (12.5%)		2 (6.1%)	2 (7.4%)	0.813
	Others	1 (12.5%)	2 (25.0%)		7 (21.2%)	5 (18.5%)	0.972
MRONJ stage (N, %)	1	3 (37.5%)	4 (50.0%)		7 (21.2%)	5 (18.5%)	0.377
	2	5 (62.5%)	3 (37.5%)		19 (57.6%)	19 (70.4%)	
	3	0	1 (12.5%)		7 (21.2%)	3 (11.1%)	

^*^p < 0.05

^**^Tooth related: tooth extraction, localized periodontitis

^***^Dental implant: dental implant insertion, implant removal, and peri-implantitis

MRONJ, medication-related osteonecrosis of the jaw

### Comparison of treatment period and number of surgical procedures

**Fig. 2:**
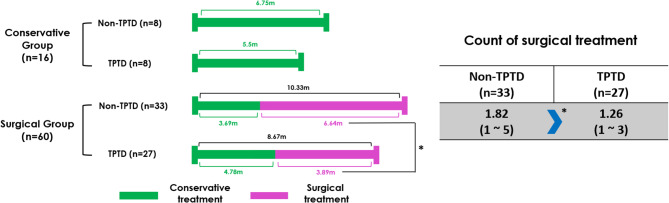
Conservative and surgical period from the first visit to treatment completion in each group and count of surgical treatments. m = month

The average treatment times for the conservative group with non-TPTD and TPTD patients were 6.75 ± 6.07 months and 5.5 ± 1.77 months, respectively, showing a shorter duration for the TPTD group than for the non-TPTD group, although not statistically significant (*p* = 0.591). In the surgical group, the average treatment times were 10.33 months and 8.67 months for non-TPTD and TPTD patients, respectively. The treatment period was shorter in the TPTD group than in the non-TPTD group; however, the difference was not statistically significant (*p* = 0.507). The period from surgery to complete recovery was significantly shorter in the TPTD group than in the non-TPTD group (TPTD, 3.89 ± 2.71 months; non-TPTD, 6.64 ± 6.38 months; *p* = 0.030). The number of surgical procedures performed was lower in the TPTD group than in the non-TPTD group (TPTD, 1.26; non-TPTD, 1.82; *p* = 0.049) (Fig. 2).

### Comparison of changes in bone marker levels before and after treatment

**Fig. 3:**
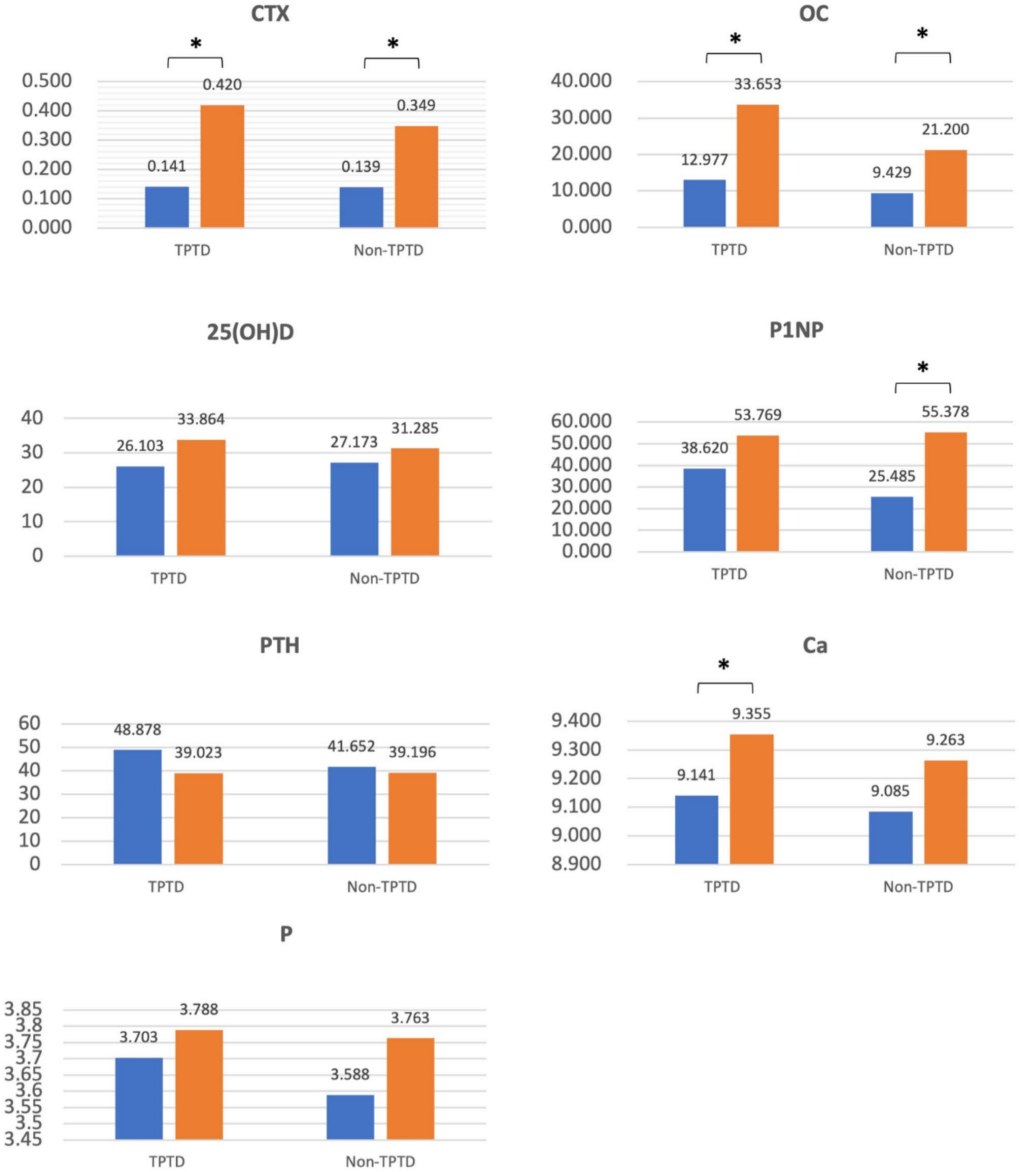
Change in bone turnover markers between TPTD and non-TPTD groups at the initial examination and MRONJ resolution (blue column, initial examination; orange column, MRONJ resolution) ^*^p < 0.05. CTX, C-telopeptide of type I collagen (ng/mL); OC, osteocalcin (ng/mL); P1NP, procollagen type 1 N-terminal propeptide (ng/mL); 25(OH)D, 25-hydroxyvitamin D (ng/mL); PTH, parathyroid hormone (pg/mL); Ca, calcium (mg/dL); P, inorganic phosphorous (mg/dL): MRONJ, medication-related osteonecrosis of the jaw; TPTD, teriparatide

The average bone marker values at the initial and final visits were summarized for both the TPTD (*n* = 35) and non-TPTD (*n* = 41) groups (Fig. 3). CTX, OC, P1NP, 25-OH-vitamin D, calcium, and inorganic P levels increased from the first visit in both groups, with only PTH levels decreasing. Post-treatment, all bone markers, except OC, showed no significant differences between the groups. However, OC levels were higher in the TPTD group than in the non-TPTD group (33.653, *p* = 0.012). Paired-sample analysis revealed significant increases in CTX, OC, and 25-OH-vitamin D levels in both groups. CTX and OC levels showed greater increases in the TPTD group than in the non-TPTD group, whereas 25-OH-vitamin D levels showed a slightly higher increase. P1NP levels increased in both groups but were significantly increased only in the non-TPTD group. PTH levels decreased similarly in both groups but without statistical significance. Calcium levels were significantly increased in the TPTD group, with no significant change in inorganic P levels.

**Fig. 4:**
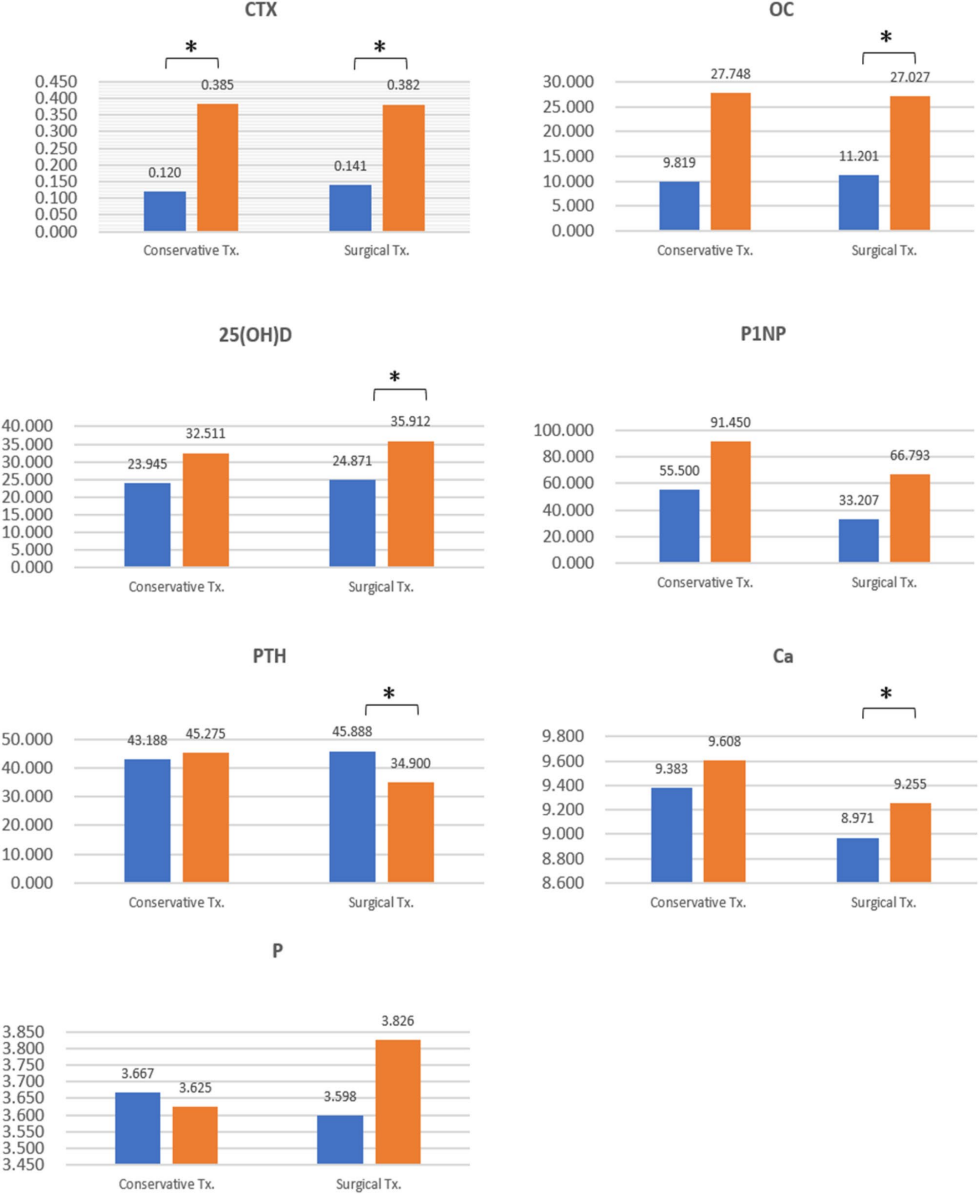
Change in bone turnover markers between conservative and surgical groups at the initial examination and MRONJ resolution (blue column, initial examination; orange column, MRONJ resolution). ^*^p < 0.05. CTX, C-telopeptide of type I collagen (ng/mL); OC, osteocalcin(ng/mL); P1NP, procollagen type 1 N-terminal propeptide (ng/mL); 25(OH)D, 25-hydroxyvitamin D (ng/mL); PTH, parathyroid hormone (pg/mL); Ca, calcium (mg/dL); P, inorganic P (mg/dL); MRONJ, medication-related osteonecrosis of the jaw

Additionally, average bone marker values at the initial and final visits were summarized for both the conservative treatment (*n* = 16) and surgical treatment (*n* = 60) groups (Fig. 4). CTX, Osteocalcin, P1NP, 25-OH-Vitamin D, and Calcium increased in both the conservative treatment group and the surgical group compared to the first visit, while parathyroid hormone levels decreased in the surgical treatment group and inorganic P decreased in the conservative treatment group. In the paired-sample analysis method, CTx was significantly increased in both treatment groups. However, OC, 25-OH-Vitamin D, and calcium were significantly increased only in the surgical treatment group. Parathyroid hormone levels were significantly decreased in the surgical treatment group.

### Comparison of T-score changes before and after MRONJ treatment in TPTD and non-TPTD groups

No significant differences in T-scores were observed before MRONJ treatment between the TPTD and non-TPTD groups (lumbar, femur neck, hip T-scores; *p* = 0.552, 0.341, 0.725, respectively). The changes in T-scores before and after MRONJ treatment were analyzed, with a focus on the lumbar spine and femur neck in the TPTD group (Table 2). In the TPTD group (*n* = 35), significant improvements were observed in both the lumbar spine and femur neck T-scores. The lumbar spine T-score improved from − 2.47 ± 0.91 to − 1.76 ± 0.89 (*p* = 0.004), and the femur neck T-score improved from − 2.47 ± 0.82 to − 2.25 ± 0.75 (*p* = 0.035).

**Table 2:** Comparison of T-score changes before and after MRONJ treatment in TPTD and non-TPTD groups

	TPTD group (*n* = 35)			Non-TPTD group (*n* = 41)		
T-score (mean ± SD)	Initial	MRONJ resolution	**p-value**	Initial	MRONJ resolution	**p-value**
Lumbar	-2.47 ± 0.91	-1.76 ± 0.89	0.004*	-2.30 ± 1.17	-2.04 ± 1.23	0.365
Femur neck	-2.47 ± 0.82	-2.25 ± 0.75	0.035*	-2.29 ± 0.68	-2.27 ± 0.72	0.733
Hip	-1.43 ± 0.99	-1.34 ± 0.64	0.378	-1.22 ± 0.69	-1.29 ± 0.68	0.465

^*^p < 0.05 (Paired T-test); MRONJ, medication-related osteonecrosis of the jaw

There was no significant difference in T-scores before MRONJ treatment between the TPTD and non-TPTD groups (lumbar, femur neck, hip T-scores; *p* = 0.552, 0.341, and 0.725, respectively)

In contrast, the non-TPTD group (*n* = 41) showed no significant changes in the lumbar spine and femur neck T-scores. These results suggest that TPTD treatment may be associated with significant improvements in bone density, particularly in the lumbar spine and femur neck, compared to the non-TPTD group.

## Discussion

MRONJ is a notable complication in patients taking antiresorptive osteoporosis drugs [3]. Its prevalence is approximately 0.02%; however, it attracts significant research attention because of its serious complications and limited treatment options [5, 19]. The 2022 AAOMS report considers both nonsurgical and surgical treatment as treatment options for MRONJ [4]. However, recent studies have placed more emphasis on surgical intervention [20, 21]. Predicting MRONJ treatment outcomes through pre-treatment serological testing and managing MRONJ-inducing drugs are key considerations [22, 23]. In this study, patients diagnosed with MRONJ received conservative treatment, including intraoral disinfection and antibiotics. Simultaneously, bone marker tests were conducted, and internal medicine consultations were held to alter MRONJ-related drugs. Surgical intervention was performed in patients with extensive bone destruction or no clinical improvement after conservative treatment. This study found a higher need for surgery (*n* = 60) than for recovery with conservative methods alone (*n* = 16), highlighting the importance of surgical techniques in MRONJ management.

Our analysis indicates that the location of MRONJ onset significantly influences treatment outcomes. The conservative group demonstrated a higher recovery rate in cases involving the maxillary posterior teeth than the surgical group, which had more instances involving the mandibular posterior teeth. However, previous studies have reported MRONJ in various locations in the maxilla and mandible, both in the anterior and posterior regions [24, 25]. Therefore, determining the incidence of MRONJ by location based on the results of this study alone is difficult.

In MRONJ treatment, alongside surgical procedures, adjuvant therapies have been extensively studied [7]. TPTD is a recombinant protein of PTH, an anabolic agent that acts directly on bone tissue, stimulating bone formation and increasing bone remodeling activity [2]. It was approved by the US Food and Drug Administration in 2002 as a therapy for osteoporosis [26]. Experiments predicting the effect of TPTD in animal MRONJ models have reported that TPTD demonstrated a certain effect [27, 28]. In the treatment of MRONJ, several studies have been conducted comparing the treatment effects of groups that administered TPTD and those that did not, and the group that was administered TPTD showed better effects than the group that was not administered TPTD [15, 29, 30]. In one study, when looking at the changes 6 months after starting MRONJ treatment, the TPTD treatment group showed better recovery than the non-TPTD group [29], and in another study, 75.9% of the patients in the TPTD treatment group showed good treatment [15]. However, there is a lack of comparative studies on surgical techniques and treatment durations in relation to TPTD use. Our study found that the TPTD group underwent fewer surgical procedures, averaging 1.26 procedures compared with 1.82 in the non-TPTD group.

The TPTD group experienced a shorter treatment period (5.5 months) than the non-TPTD group (6.75 months). Similarly, in the surgical group, the TPTD subgroup showed a reduced treatment period (8.67 months vs. 10.33 months), but these differences were not statistically significant. From the start of the surgical intervention to recovery, the TPTD group took an average of 3.89 months, significantly lesser than the 6.64 months in the non-TPTD group, indicating that TPTD can potentially shorten the treatment period when combined with surgery. This aligns with the findings that TPTD effectively separates necrotic from vital bone, potentially reducing the treatment duration and surgical interventions. According to Doh et al., the administration of TPTD is effective in separating necrotic bone from the surrounding vital bone [31], and this action can reduce the MRONJ treatment time and surgical procedures.

Drugs that cause MRONJ are also commonly used in patients with osteoporosis. In this study, we confirmed that the T-scores were significantly reduced in the TPTD group than in the non-TPTD group. Patients with MRONJ who received TPTD treatment are generally undergoing active treatment for osteoporosis, even during the withdrawal period from bisphosphonate or denosumab. Through TPTD treatment, we believe osteoporotic complications such as osteoporotic fractures during the withdrawal period can be reduced [32, 33]. 

In MRONJ treatment, changes in bone marker patterns following the discontinuation or substitution of osteoporosis drugs and during TPTD treatment are crucial for assessing bone remodeling. CTX, which is derived from a protein matrix, serves as a resorption marker, whereas OC and P1NP, which are produced by osteoblasts, are involved in bone formation [12, 34, 35]. Studies have indicated increased levels of these markers after TPTD injection, suggesting enhanced bone metabolism [19, 31, 36, 37]. In our study, both the TPTD and non-TPTD groups showed increased levels of these markers, with the TPTD group exhibiting significantly higher values of CTX and OC than the non-TPTD group. P1NP levels increased in both groups, but a significant difference was noted only in the non-TPTD group, possibly because fewer P1NP tests were performed in the TPTD group.

Vitamin D levels, measured by 25-OH-vitamin D tests, play a vital role in bone metabolism [29]. Vitamin D deficiency is associated with an increased incidence of MRONJ [38]. Our study found significant increases in 25-OH-vitamin D levels in both groups. However, other studies have reported conflicting results during TPTD treatment, with a decrease in 25-OH-vitamin D levels and an increase in 1,25-OH-vitamin D levels [39].

PTH, regulating calcium and phosphorus metabolism and stimulating bone formation, is crucial in osteoporosis treatment [40]. Long-term bisphosphonate use reduces PTH levels [41]. In this study, despite the discontinuation or substitution of osteoporosis drugs during MRONJ treatment, PTH levels decreased in both groups without significant differences, indicating a need for further studies on MRONJ treatment and PTH level changes.

Levels of calcium and phosphorus, which are key components of bone composition, were also studied. Low blood calcium levels are associated with a higher MRONJ risk [42], and our study showed a significant increase in calcium levels only in the TPTD group, suggesting a potential reduction in MRONJ risk. However, the risk of hypercalcemia after TPTD treatment warrants caution [43]. The relationship between inorganic phosphorus levels and MRONJ remains underexplored, calling for more studies.

The study findings on the differences in treatment duration and number of surgeries between the TPTD and non-TPTD groups highlight the significance of TPTD in MRONJ treatment. The shorter treatment period and fewer surgeries in the TPTD group, along with positive results from bone marker evaluations, confirmed the effectiveness of TPTD as an adjunct treatment in MRONJ management.

Although this study underscores the efficacy of TPTD in the treatment of MRONJ, its limitations warrant further consideration. First, the sample size was limited, potentially affecting the generalizability of the findings. The retrospective design of the study may not adequately account for all confounding variables possibly influencing the outcomes. Additionally, the focus on quantitative measures, such as treatment duration and bone marker levels, overlooks the qualitative aspects of patient recovery, such as pain management and post-treatment quality of life, which are crucial for a comprehensive understanding of treatment efficacy.

If more extensive surgical procedures, such as wide resections, had been employed from the onset, the recurrence rate might have been lower, potentially reducing the number of surgical interventions required. This indicates that surgical approach itself may have played a more significant role in influencing outcomes than the administration of TPTD. These findings suggest that further research is necessary to fully understand the interplay between surgical techniques and adjunctive therapies like TPTD in the treatment of MRONJ.

Furthermore, although TPTD’s benefits in reducing treatment time and surgical needs are highlighted in the study, its long-term effects and potential adverse reactions across different patient demographics have not been thoroughly investigated. This gap highlights the need for ongoing in-depth studies to understand the full scope of the effect of TPTD on MRONJ treatment, particularly over prolonged periods and in various patient groups. In summary, this study significantly contributes to the knowledge of TPTD’s role in MRONJ treatment and emphasizes the need for further studies to refine MRONJ management strategies.

## Conclusions

This study demonstrates that TPTD’s application during MRONJ treatment reduces the surgical period and number of procedures, demonstrating its efficacy as an adjunct therapy. It also reveals that TPTD-treated patients exhibit not only higher increases in the levels of crucial bone markers, such as CTX, OC, 25-OH-vitamin D, and calcium, but also significant improvements in bone density, particularly in the lumbar spine and femur neck as indicated by T-score changes. This suggests enhanced bone metabolism and a greater potential for more effective MRONJ treatment. However, this study has certain limitations, such as a limited sample size and retrospective design. It also highlights the need for further studies including long-term out-come to better understand the role of TPTD and optimize its use in the management of MRONJ.

## Data Availability

The datasets used and/or analyzed during the current study are available from the corresponding author on reasonable request.

## Abbreviations

TPTD: Teriparatide
MRONJ: Medication-related osteonecrosis of the jaw
CTX: Type 1 collagen
OC: Osteocalcin
P1NP: Procollagen type 1 N-terminal propeptide
PTH: Parathyroid hormone
25(OH)D: 25-OH-vitamin D
AAOMS: American association of oral and maxillofacial surgeons
DXA: Dual-energy X-ray absorptiometry
